# Effects of Active Video Games on Children’s Psychosocial Beliefs and School Day Energy Expenditure

**DOI:** 10.3390/jcm8091268

**Published:** 2019-08-21

**Authors:** Zan Gao, Zachary C. Pope, Jung Eun Lee, Minghui Quan

**Affiliations:** 1 Department of Sport Rehabilitation, School of Kinesiology, Shanghai University of Sport, Shanghai 200438, China; 2 School of Kinesiology, University of Minnesota-Twin Cities, Minneapolis, MN 55455, USA; 3 School of Public Health, University of Minnesota-Twin Cities, Minneapolis, MN 55455, USA; 4 Department of Applied Human Sciences, University of Minnesota, Duluth, MN 55812, USA

**Keywords:** active video games, outcome expectancy, pediatric obesity, self-efficacy, social support

## Abstract

**Purpose:** Examine the effects of active video games (AVGs) on children’s school-day energy expenditure (EE) and physical activity (PA)-related self-efficacy, social support, and outcome expectancy over 9 months. **Method:** Participants were 81 fourth grade students (${\bar{X}}_{\mathrm{age}}$ = 9.23 years, SD = 0.62; 39 girls) from two urban Minnesota elementary schools. A once-weekly 50 min AVG intervention was implemented in the intervention school for 9 months in 2014–2015 while the control school continued regular recess. Children’s school-day EE (daily caloric expenditure) and mean daily metabolic equivalent (MET) values were estimated via accelerometry whereas self-efficacy, social support, and outcome expectancy were assessed with psychometrically-validated questionnaires. All measures were completed at baseline and at the 4th and 9th months. **Results:** We observed significant interaction effects for daily caloric expenditure, *F*(1, 58) = 15.8, *p* < 0.01, mean daily MET values, *F*(1, 58) = 11.3, *p* < 0.01, and outcome expectancy, *F*(1, 58) = 4.5, *p* < 0.05. Specifically, intervention children had greater increases in daily caloric expenditure (91 kilocalorie/day post-intervention group difference), with control children decreasing daily caloric expenditure over time. We observed identical trends for mean daily MET values (0.35 METs/day post-intervention group difference). Interestingly, we observed outcome expectancy to increase in the control children, but decrease among intervention children, at post-intervention (1.35 group difference). Finally, we observed a marginally significant interaction effect for social support, *F*(1, 58) = 3.104, *p* = 0.08, with an increase and decrease seen in the intervention and control children, respectively. We observed no interaction or main effects for self-efficacy. **Discussion:** Observations suggested an AVG intervention contributed to longitudinal increases in school-day EE and social support compared to the control condition. Future research should examine how self-efficacy and outcome expectancy might be promoted during school-based AVG interventions.

## 1. Introduction

Pediatric obesity continues to be a substantial public health burden in the US [1]. The obesity epidemic and subsequent physiological and psychosocial wellbeing of low-income and ethnically diverse children have become particularly concerning, partially due to the physical inactivity and sedentary behavior rates commonly observed in this population [2]. For example, children from underserved families of low socioeconomic status (SES) are more likely to be overweight and/or obese than children from middle- to upper-SES families [3]. As a result, these children are more likely to develop cardiovascular disease, type II diabetes, and other chronic diseases [4]. Regular physical activity (PA) participation has been evidenced to prevent and reduce pediatric obesity [5,6]. Thus, it is imperative to develop innovative technology-based interventions to promote PA participation and curb pediatric obesity among children of low SES living in underserved urban areas.

### 1.1. Application of Active Video Games in Physical Activity Promotion

As known, technologies such as computers and video games have contributed tremendously to sedentary behavior and physical inactivity. For example, traditionally sedentary video games and/or computer video games have been blamed for players’ sedentary lifestyles and have played a significant role in the epidemic of pediatric obesity [7]. On the other hand, many newly emerging technologies, including active video games (AVGs; also known as exergaming), virtual reality, and smart bands, have been increasingly utilized for physical activity and health promotion in children and adults alike [8].

Briefly, AVGs refer to a genre of movement-based video games that are also a form of exercise [9]. Despite the negative impact of sedentary video games on obesity, AVGs have the potential to help promote a healthy lifestyle in youth while also assisting in fighting pediatric obesity [10,11,12,13]. In the past decades, the fast growth of AVGs have led to the development of new interactive exercise strategies, with the quality of field-based AVG interventions improving dramatically over this time period [14,15,16,17]. Recently, AVGs have been increasingly used within schools as an innovative and fun approach for promoting a physically active lifestyle in children and adolescents [18,19,20], with this research often investigating the benefits of AVGs as a means for increasing PA. Short-term field and laboratory studies have indeed suggested that AVGs can improve children’s PA levels [20,21,22], cardiovascular fitness, and psychosocial outcomes [23], in addition to reducing weight [24]. However, these investigations have primarily been conducted in children from families of middle and upper SES.

Empirical work examining the long-term effects of AVGs on the physiological health outcomes of children from low SES families living in urban areas in field-based settings has been scarce. Moreover, most previous AVG studies also only focused on one health outcome/domain (e.g., effect of Dance Dance Revolution/Wii on energy expenditure (EE)). Studies are warranted, however, examining the effects of AVGs on other outcomes important to childhood development, such as psychosocial beliefs, with researchers having recommended conducting PA interventions which influence psychosocial constructs that have been observed as key determinants of PA behavior change [25,26,27].

### 1.2. Theoretical Framework

The Social Cognitive Theory (SCT) [28,29] has been extensively utilized to intervene and promote changes to children’s PA behavior over the past decade. The SCT proposes that behavioral variability results from the reciprocally determinant interaction between the performance of the behavior (e.g., PA behavior), environmental factors (e.g., social environment), and personal factors (e.g., beliefs, cognition, motivation). Given AVGs’ enjoyable and easy-to-learn nature, as well as AVGs’ ability to be played as group, the SCT-related psychosocial variables of self-efficacy, outcome expectancy, and social support may be able to be promoted using this technological platform if incorporated into children’s daily routine—offering the potential to promote long-term PA participation.

### 1.3. Purpose Statement and Research Hypotheses

Children spend most of their waking hours at school. Yet, while several empirical AVG interventions in youth have been conducted in extracurricular settings [20,23], few interventions have been incorporated into school curricular. Further, as briefly noted, studies of AVG interventions in youth have often been short in duration and limited in the outcomes accessed, with an additional limitation being that these studies used a single gaming platform as opposed to using multiple gaming platforms which might assist in keeping children engaged throughout the intervention’s duration. To build upon previous studies’ limitations, this study investigated the effects of a 9 month school-based AVG intervention on the EE and psychosocial beliefs of children from low SES families living in underserved urban areas in Minnesota. The following two hypotheses were proposed: (1) children in the AVG intervention group would demonstrate greater increases in EE and PA intensity than those in the control group; and (2) children in the AVG intervention group would demonstrate greater improvements in psychosocial beliefs (self-efficacy, outcome expectancy, and social support) than those in the control group.

## 2. Methods

### 2.1. Participants and Research Design

We calculated sample size using G*Power 3.1 [30] which indicated that 40 participants would be sufficient for 80% power (*α* = 0.05, effect size = 0.30) to test the primary outcome (i.e., EE). In total, we recruited 81 fourth grade children, aged 9 to 10 years old, from two urban elementary schools in the US state of Minnesota. The majority of the participants were African American (54.3%), with 89% of these children coming from economically disadvantaged families (receive free/reduced meals). Full demographic characteristics are shown in Table 1. Specific inclusion criteria for children to participate in this study were: (1) enrollment at an urban public Title I school (meaning ≥50% of children received free or reduced-price breakfast and lunch); (2) aged 9–10 years; (3) without a diagnosed physical or mental disability according to school records; and (4) provided parental consent and child assent. Prior to the study, parental/guardian consent and child assent were obtained in compliance with the standards of the Institution (Institution Review Board code: 1301M26882) and school district research committee as well as the 1964 Helsinki Declaration and its later amendments or comparable ethical standards.

We used a two-arm, 9 month experimental design with repeated measures. Importantly, the curricular policies for the two target schools were identical and required a 50 min weekly physical education class and a daily 20 min recess. With these curricular policies in mind, we designated schools as the experimental unit and assigned the schools to one of two groups: (1) Intervention Group: children engaged in one 50 min AVG session/week beyond time spent in physical education; and (2) Control Group: children did not engage in any AVGs play nor any other structured school-based PA programs beyond physical education. Participants’ baseline EE and psychosocial beliefs were assessed at the beginning of school year, with these outcomes assessed again at the 4th and 9th months of the school year (detailed below).

### 2.2. Intervention

*AVG Intervention Condition.* With the school administrators’ support, we established a weekly AVG program in the intervention school. The program was integrated into the intervention school’s curriculum, with a full-time physical education teacher supervising the program. Specifically, we set up eight AVG stations in a fitness room, with each station equipped with one of two AVG systems (Xbox 360 (Microsoft; Redmond, WA, USA) or Nintendo Wii (Nintendo; Kyoto, Japan)), a TV, and necessary ancillary supplies. We offered several developmentally-appropriate AVGs including: Just Dance, Wii Fit, Gold’s Gym Cardio Workout, and Kinect Sports. Each station accommodated the gameplay of up to four children, with children rotating from one station to another station every 10 min—allowing for a 45 s transition. The multiple platforms and rotating sequence allowed all children in one class the opportunity to play AVGs in a group setting while also being able to play a variety of AVGs during the program—helping reduce boredom over the intervention’s duration. School teachers recorded intervention children’s attendance in the program.

*Control Condition.* Children in the control school continued their regular recess activities which included non-structured play on an outdoor playground or within a classroom or gym (depending on the weather). Classroom teachers supervised recess, but no structured PA was offered during this period aside from the children’s weekly physical education class. During recess, children engaged in a variety of activities including: games of chase, tag, four square, and basketball in addition to playing on other playground equipment (e.g., swings). Recess duration remained constant throughout the school year, as aforementioned, the control school had identical physical education and recess duration compared to the intervention school.

*Intervention Fidelity.* We continuously monitored intervention fidelity for the AVG intervention program. In particular, we met regularly with the school teachers for program training and monitoring to ensure the consistency of the intervention program and monitored the AVG intervention implementation on a weekly basis with previously established protocols [31]. These procedures allowed us to determine whether all AVG intervention protocol elements were covered during each AVG session. Finally, we also collected process evaluation surveys to further monitor implementation as well as noting content fidelity and child engagement. In the present study, intervention fidelity exceeded 90% for the protocol elements during the AVG sessions. In terms of the regular recess at the control school, we requested the school teachers offer the usual practice as they did in the past and monitored the program on a regular basis.

### 2.3. Measures

*Energy Expenditure.* We estimated children’s EE and kilocalories/day during all school hours using ActiGraph GT3X+ accelerometers (ActiGraph Ltd., Pensacola, FL, USA). ActiGraph accelerometers have been evidenced as valid and reliable when assessing children’s EE and PA [32,33]. We placed the accelerometers on children’s right hip at the level of the superior iliac crest using an elastic belt. To estimate PA intensity and EE, we used the Evenson et al. (2008) cut points (in counts/minute; sedentary: 0–100; Light PA: 101–2295; and moderate-to-vigorous PA: ≥2296) [32]. Following standardized monitor wearing protocols, children wore the accelerometers for 3 days to capture their regular PA behavior at baseline and at the 4th and 9th months [33]. Both outcome data (daily metabolic equivalents (METs) and kilocalories/day) were validated within ActiLife (v. 6.13; ActiGraph Corp, Pensacola, FL, USA) and truncated to match the children’s school day (6 h 20 min).

*Psychosocial Beliefs.* We assessed select SCT-related psychosocial beliefs (self-efficacy, outcome expectancy, and social support) using established questionnaires validated in children and adolescents [34,35,36,37]. In detail, a six question survey assessed children’s self-efficacy on a seven point Likert-type scale (1 = I am very sure I can’t to 7 = I am very sure I can), with children responding to the stem “Rate how sure you are that you can do PA in each situation” [34]. A nine question survey assessed outcome expectancy on an adapted 10 point Likert-type scale (1 = very unlikely to 10 = very likely), with the sample question on this survey being “If I were to exercise most days it would…” [36]. Finally, to assess children’s social support, we used an 11 question survey with a five point Likert-type scale (1 = hardly ever or never to 5 = everyday) [37]. This survey questioned children regarding the support and encouragement they receive for PA from parents, teachers, and friends. The mean scores of the surveys were used as children’s outcomes for self-efficacy, outcome expectancy, and social support.

*Demographic and Anthropometric Information.* We assessed the participants’ demographic information along with the psychosocial beliefs survey, including their race/ethnicity, sex, age, grade. Participants’ height was also assessed to the nearest half-centimeter with the Seca Stadiometer (Seca; Hamburg, Germany) and their weight was assessed to the nearest tenth of a kilogram via the Detecto weight scale (Detecto; Webb City, MO, USA). We also calculated their body mass index (BMI) by dividing weight in kilograms by height in meters, followed by the calculation of the BMI percentiles based on sex and age.

### 2.4. Procedures

Baseline data were collected over a 2 week period. During the first week, we introduced the study and obtained parental consent forms and child assent forms. During the second week, we explained to the children how to complete the demographic information sheet and assessed their height and weight at a separate, private room. Children’s EE during the school day was then measured for three consecutive days using ActiGraph GT3X+ accelerometers and their psychosocial beliefs evaluated within the classroom in coordination with the teachers’ curricular schedules. Following the second week, we implemented the AVG program at the intervention school. We used identical data collection procedures at the 4th and 9th months. It is important to note that no AVG intervention was offered during the data collection periods for EE.

### 2.5. Data Analysis

Data were imported from Excel into SPSS 23.0 (IBM Inc.; Armonk, NY, USA) for descriptive and inferential statistical analyses. We first screened for non-normality using Shapiro–Wilks tests and examined these data for outliers. Cronbach’s alpha coefficients were then calculated to assess the internal consistency of the psychosocial belief surveys. Next, we conducted a descriptive analysis to describe the sample characteristics, with frequencies or means ± standard deviation reported, as appropriate, for all demographic, anthropometric, physiological, and psychosocial outcomes. Given the fact that participants of these two schools differed significantly in light of age and race/ethnicity, we conducted one-way (between-subjects factor: group) Multivariate Analysis of Covariance with repeated measures to examine if children’s daily METs and daily caloric expenditure differed between the two schools across time (within-subjects factor: time), with age and race/ethnicity as the covariates. The same statistical analysis approach was employed for the three psychosocial beliefs. Significance was set at *p* ≤ 0.05 for all inferential analyses, with effect sizes reported for each comparison. We used partial eta-squared (η^2^) as an index of effect size, for which small, medium, and large effect sizes were designated as 0.10 ≤ 0.25, 0.25 < 0.40, and ≥0.40, respectively [38].

## 3. Results

*Descriptive Analyses.* After date screen, the final sample was comprised of 81 children (${\bar{X}}_{\mathrm{age}}$ = 9.23 ± 0.62; 39 girls), with the sample’s ethnic distribution primarily African American (*n* = 44) followed by White American (*n* = 25). The remaining 12 participants were from other ethnicities including Hispanic, Native, and Asian Americans. Cronbach’s alpha coefficients indicated the psychosocial belief surveys exceeded 0.70 (0.72–0.85 for three rounds of assessment) and suggested that the surveys had acceptable internal consistency values for use in this population [39]. Descriptive statistics are reported in Table 1. At baseline, we noted that the children in the control group were younger and of shorter height compared to children in the intervention group. Additionally, the intervention group had significantly more African American children than the control group.

*Group Differences.* On average, children in the intervention group had increased METs/day whereas children in the control group demonstrated decreased METs/day over time (See Table 2). Both groups’ daily caloric expenditure increased markedly across time, but the intervention children demonstrated significantly greater increases in this outcome. Meanwhile, children displayed moderate to high levels for all psychosocial beliefs assessed, as all mean scores of these outcomes were greater than the median values of the scales. Regardless of groups, children demonstrated increases improvement in psychosocial beliefs over time.

In light of inferential analyses, there were 63 participants with completed EE data and 68 participants with completed data for psychosocial beliefs. Our preliminary analysis indicated that there was no significant differences on the demographic variables between those with completed data and those who did not provide completed data. MANCOVA with repeated measures for EE yielded significant effect, Wilks’ Lambda = 0.55, *F*(4, 56) = 11.61, *p* < 0.01, η^2^ = 0.45. In particular, we observed significant group by time interaction effects for mean daily METs, *F*(1, 59) = 6.35, *p* < 0.01, η^2^ = 0.10; and daily caloric expenditure, *F*(1, 59) = 10.90, *p* < 0.01, η^2^ = 0.16. Specifically, intervention children had greater increases in mean daily METs over time while the control children demonstrated decreases in mean daily METs—resulting in a 0.35 METs/day post-intervention group difference. Given the higher mean daily MET values in the invention group, we observed identical trends for daily caloric expenditure, with intervention children demonstrating a 91 kilocalorie/day higher value at post-intervention compared to the control children. MANCOVA with repeated measures for psychosocial beliefs also revealed significant effect, Wilks’ Lambda = 0.84, *F*(4, 61) = 3.00, *p* = 0.03, η^2^ = 0.16. Interestingly, we noted that control children had increased outcome expectancy at post-intervention while the intervention children demonstrated decreased values over time (1.35 group difference). Finally, we observed a marginally significant group by time interaction effect for social support, *F*(1,64) = 2.74, *p* = 0.08, η^2^ = 0.06, with an increase and decrease seen in the intervention and control children, respectively. In addition, there were significant group main effects for mean daily METs/day, *F*(1,59) = 11.14, *p* < 0.01, η^2^ = 0.16, kilocalories/day, *F*(1,59) = 17.38, *p* < 0.01, η^2^ = 0.23, and outcome expectancy, *F*(1,64) = 2.80, *p* < 0.05, η^2^ = 0.10. No interaction or main effects were observed for self-efficacy.

## 4. Discussion

AVGs have been increasingly integrated into various field-based programs due to this technology’s potential to promote PA and health in children—perhaps assisting in efforts to curb the current pediatric obesity epidemic [16,17,18,22,23,40,41,42,43,44]. In the present study, we examined if children in an underserved urban school included in an AVG intervention would demonstrate greater increases in EE and improvements in SCT-related psychosocial beliefs (self-efficacy, outcome expectancy, and social support) compared to a control group within a second underserved school over the course of one 9 month academic year. Our observations suggested that an AVG intervention may be able to promote improvements in EE and social support but that other strategies may be necessary to increase outcome expectancy and self-efficacy during an AVG intervention.

Our first hypothesis proposed that children participating in the AVG intervention would have greater increases in EE than those in the control group. We confirmed this hypothesis as the intervention children showed significantly greater increases in daily caloric expenditure versus the control children over 9 months—likely resultant from the higher mean daily METs observed in the intervention children (i.e., higher mean PA intensity throughout the day). These observations corroborate previous research suggesting AVGs have potential to improve children’s PA and EE [4,31,43,45,46,47] in addition to literature which has noted AVGs to stimulate higher overall PA levels compared to usual recess activities among children [48,49,50]. For example, Gao et al. found that children in an AVG intervention demonstrated higher EE and MVPA than children in a control group [31]. Importantly, in a sample of minority children of low SES similar to the current study, a 6 week AVG intervention indicated that AVGs might be a feasible approach to promote PA [51]. Improvements in daily EE and mean METs might be attributed to children’s known enjoyment of AVG-based activities observed in past literature [17,22,41,42]. Indeed, AVGs can capitalize on children’s interest in video and computer interaction by integrating interactive exercise equipment and technology to get children moving [41,42,44]. Notably, we ensured that the AVG program in the current study had multiple gaming stations and various games to prevent boredom over the 9 month intervention period. This design might have boosted children’s enjoyment and subsequently promoted increased PA, and subsequent EE, at school over time. As children from underserved families of low SES are more likely to be physically inactive and develop obesity/other chronic diseases in adulthood compared to their middle- to upper-class counterparts [5,6,7], our results are encouraging. Specifically, our observations suggested that an AVG intervention may be a feasible strategy to boost EE levels in children from underserved urban schools over a 9 month period—a population which typically has less PA opportunities.

Our second research hypothesis postulated that children included in the AVG intervention would report significantly greater improvements in SCT-based psychosocial beliefs (self-efficacy, outcome expectancy, and social support) than control children. We observed partial support for this hypothesis as the intervention children had greater increases in perceived social support over time as compared to control children, and the inferential statistics approached significance level. According to literature, the impact of various PA interventions on children’s perceived social support has been mixed. For instance, Gao et al. reported that children in an intervention group who participated in an interactive dancing video game had greater increases in social support versus the comparison children [18]. Yet, other investigations have reported no differences for changes in social support over time between intervention and control groups [52] or decreased social support from baseline to post-intervention in the intervention group versus control [53]. These inconclusive observations may be due to differences in the sample studied, the research design, the nature of PA intervention, the intervention length, and intervention fidelity. In fact, researchers have posited that friend support is more important for adolescents and young adults whereas the support of teachers and parents is more important for children [54,55,56]. Given the fact that the social support we employed in the present study was a combination of teacher support, family support, and friend support, we may not have seen as great a change as Gao et al. observed [18]. On the other hand, we assigned intervention children to eight AVG stations in the current study, with up to four children playing simultaneously at each station. Under such circumstances, these children had more opportunities to interact with their friends and teachers [18]. Thus, when compared to the regular recess activities of the control group, the cooperative gaming environment likely fostered greater social support from significant others (e.g., friends, teachers) for intervention children—producing changes in this outcome in the expected direction.

AVG interventions have been evidenced to exert positive effects on children’s self-efficacy [18,22]. Gao and colleagues, working with 53 fourth grade children from an urban community, found that children reported significantly higher scores for self-efficacy and enjoyment during an AVG intervention compared to aerobic dance [22]. In a longitudinal study also by Gao et al. [18] an AVG intervention again had a positive effect on upper elementary school children’s self-efficacy across one 9 month academic year. It was hypothesized that the preceding reported increases in self-efficacy were resultant from the tutorial learning models employed to learn how to play the AVGs as well as the instant feedback AVGs provided during gameplay. Yet, despite the similar methodology used in this study, children’s self-efficacy did not differ between groups over time. The specific reasons for this observation are unknown based upon our survey data. Future research should explore how AVG-related self-efficacy might be promoted in this population through qualitative methodology (e.g., individual interviews, focus groups).

Contrary to our hypothesis, we observed control children to demonstrate greater increases in outcome expectancy over time than intervention children. Targeting a similar population, Gao et al. implemented a dance-based AVG intervention and found no differences in outcome expectancy between the intervention and comparison group [18]. In fact, these researchers noticed that the comparison children had slightly, yet non-significantly, higher outcome expectancy scores at post-intervention verse intervention children. Taken together, we conclude that, although AVGs have some advantages in PA and health promotion in younger generations, AVGs are devoid of information on the health benefits of regular PA participation. Future investigations might include supplemental lessons on the health benefits of PA when implementing an AVG intervention; thus increasing the likelihood of improving outcome expectancy. Indeed, in the present study, the control children may have become more aware of the health benefits of PA as a result of the more traditional physical education at that school versus the intervention school.

This study had several strengths including: (1) inclusion of children from ethnically diverse and underserved backgrounds; (2) long intervention period; (3) incorporation of multiple AVG stations, game consoles, and game options; (4) EE estimation using accelerometry; (5) the investigation of children’s psychosocial outcomes as a result of an AVG intervention as opposed to only physiological outcomes; and (6) routine intervention fidelity procedures. Several study limitations, however, should be noted. First, children’s EE outside of school was not assessed, so we cannot hypothesize how the AVG intervention might have affected EE during these time periods. Future studies should estimate children’s full-day EE using accelerometry. Second, the participants came from two urban schools in a midwestern metropolitan city, with group assignment occurring at the school-level to avoid data contamination. Although we controlled age and race/ethnicity as the covariates in this study, this arrangement limited the generalizability of our observations. Future interventions might be implemented using group randomized trial design with multiple school sites in underserved communities across a range of geographic areas.

## 5. Conclusions

Our observations suggested that the implementation of an AVG intervention had positive effects on children’s mean daily MET levels, daily caloric expenditure, and social support, but that this intervention was unable to promote improved self-efficacy and outcome expectancy. While improvements to the intervention model can be made, these observations provide initial evidence for the use of innovative technology-based interventions within a school setting to help improve longer-term physiological and psychosocial health outcomes of children from families of low SES living in underserved urban areas [42,57].

## Figures and Tables

**Table 1:** Characteristics of children.

Variables	Control (*n* = 45)	Intervention (*n* = 36)	*p* Value *
Age, years	9.09 (0.42)	9.42 (0.77)	0.02
Gender			0.55
Boys, number (%)	22 (48.9)	20 (55.6)	
Girls, number (%)	23 (51.1)	16 (44.4)	
Race/ethnicity			0.01
African American, *n* (%)	14 (31.1)	30 (83.3)	
Non-Hispanic American, *n* (%)	22 (48.9)	3 (8.3)	
Others, *n* (%)	9 (20.0)	3 (8.4)	
Height, cm (M/SD)	136.09 (7.53)	141.69 (6.84)	0.01
Weight, kg (M/SD)	37.02 (10.21)	39.72 (9.15)	0.22
BMI, kg/m^2^ (M/SD)	19.78 (4.21)	19.62 (3.52)	0.86
BMI (percentiles), % (M/SD)	69.32 (28.16)	70.00 (28.33)	0.85

BMI = body mass index; M = mean; SD = standard deviation. *: Student’s *t*-test for continuous variables and Chi-square test for categorical variables.

**Table 2:** Energy expenditure and psychosocial beliefs across time.

	Intervention Group			Control Group			F	*p*	η^2^
	Baseline	4th Month	9th Month	Baseline	4th Month	9th Month			
METs	1.71/0.20	1.73/0.25	1.81/0.20	1.74/0.16	1.53/0.12	1.45/0.52	11.31	0.001	0.16
Kilocalories per day	75.17/37.70	223.67/103	254.31/95.51	64.74/29.21	130.96/55.77	162.51/88.15	15.75	0.001	0.21
Self-efficacy	4.73/1.39	4.73/1.39	4.82/1.56	5.13/1.26	5.13/1.26	5.16/1.48	0.02	0.90	0.01
Outcome expectancy	7.68/1.59	6.82/1.89	6.86/2.23	8.00/1.54	7.97/1.13	8.21/1.23	4.46	0.04	0.07
Social support	4.14/1.49	4.14/1.49	4.64/1.62	4.03/1.52	4.03/1.53	3.66/1.91	3.10	0.08	0.05

Note. MET = metabolic equivalent; M/SD = Mean/Standard Deviation; F, *p*, and η^2^ values were used for the group by time interaction effects.

## References

[1] Ogden C.L., Carroll M.D., Kit B.K., Flegal K.M. (2012). Prevalence of obesity and trends in body mass index among US children and adolescents, 1999–2010. JAMA.

[2] Gordon-Larsen P., Nelson M.C., Popkin B. (2004). Longitudinal physical activity and sedentary behavior trends: Adolescence to adulthood. Am. J. Prev. Med..

[3] Ogden C.L., Carroll M.D., Curtin L.R., McDowell M.A., Tabak C.J., Flegal K.M. (2006). Prevalence of overweight and obesity in the United States (1999–2004). JAMA.

[4] Nelson M.C., Gordon-Larson P. (2006). Physical activity and sedentary behavior patterns are associated with selected adolescent health risk behaviors. Pediatrics.

[5] Gao Z. (2018). Growth trajectories of young children’s objectively determined physical activity, sedentary behavior, and body mass index. Child. Obes..

[6] Fan X., Cao Z.B. (2017). Physical activity among Chinese school-aged children: National prevalence estimates from the 2016 Physical Activity and Fitness in China-The Youth Study. J. Sport Health Sci..

[7] Gao Z., Pope Z., Zeng N., Gao Z. (2017). Foundations of technology and health effects of physical activity. Technology in Physical Activity and Health Promotion.

[8] Sun H., Gao Z., Zeng N., Gao Z. (2017). Overview: Promoting physical activity and health through emerging technology. Technology in Physical Activity and Health Promotion.

[9] Sinclair J., Hingston P., Masek M. (2007). Considerations for the design of exergames. ACM.

[10] Pasco D., Roure C., Kermarrec G., Pope Z., Gao Z. (2017). The effects of a bike active video game on players physical activity and motivation. J. Sport Health Sci..

[11] Biddiss E., Irwin J. (2010). Active video games to promote physical activity in children and youth: A systematic review. Arch. Pediatric. Adolesc. Med..

[12] Foley L., Maddison R. (2010). Use of active video games to increase physical activity in children: A (virtual) reality?. Pediatric. Exerc. Sci..

[13] Guy S., Ratzki-Leewing A., Gwadry-Sridhar F. (2011). Moving beyond the stigma: Systematic review of video games and their potential to combat obesity. Int. J. Hypert..

[14] Graf D., Pratt L., Casey H., Short K. (2009). Playing active video games increases energy expenditure in children. Pediatrics.

[15] Mcdonough D.J., Pope Z., Zeng N., Lee J., Gao Z. (2018). Comparison of college students’ energy expenditure, physical activity, and enjoyment during exergaming and traditional exercise. J. Clin. Med..

[16] Edwards J., Jeffrey S., May T., Rinehart N.J., Barnett L.M. (2017). Does playing a sports active video game improve object control skills of children with autism spectrum disorder?. J. Sport Health Sci..

[17] Gao Z., Podlog L., Huang C. (2012). Associations among children’s situational motivation, physical activity participation, and enjoyment in an interactive dance game. J. Sport Health Sci..

[18] Gao Z., Huang C., Liu T., Xiong W. (2012). Impact of interactive dance games on urban children’s physical activity correlates and behavior. J. Exer. Sci. Fit..

[19] Gao Z., Hannon J.C., Newton M., Huang C. (2011). The effects of curricular activity on students’ situational motivation and physical activity levels. Res. Q. Exerc. Sport.

[20] Quan M., Pope Z., Gao Z. (2018). Examining young children’s physical activity and sedentary behaviors in an exergaming program using accelerometry. J. Clin. Med..

[21] Maddison R., Mhurchu C.N., Jull A., Jiang Y., Prapavessis H., Rodgers A. (2007). Energy expended playing video console games: An opportunity to increase children’s physical activity?. Pedia. Exerc. Sci..

[22] Gao Z., Zhang T., Stodden D.F. (2013). Children’s physical activity levels and their psychological correlated in interactive dance versus aerobic dance. J. Sport Health Sci..

[23] Ye S., Lee J., Stodden D., Gao Z. (2018). Impact of exergaming on children’s motor skill performance and health-related fitness: A quasi-experimental study. J. Clin. Med..

[24] Mhurchu C.N., Maddison R., Jiang Y., Jull A., Prapavessis H., Rodgers A. (2008). Couch potatoes to jumping beans: A pilot study of the effect of active video games on physical activity in children. Int. J. Behav. Nutri. Phys. Act..

[25] Lewis B.A., Marcus B.H., Pate R.R., Dunn A.L. (2002). Psychosocial mediators of physical activity behavior among adults and children. Am. J. Prev. Med..

[26] Dishman R.K., Motl R.W., Saunders R., Felton G., Ward D.S., Dowda M., Pate R.R. (2004). Self-efficacy partially mediates the effect of a school-based physical activity intervention among adolescent girls. Prev. Med..

[27] Dishman R.K., Motl R.W., Saunders R., Felton G., Ward D.S., Dowda M., Pate R.R. (2005). Enjoyment mediates effects of a school-based physical activity intervention. Med. Sci. Sports Exerc..

[28] Bandura A. (1986). Social Foundations of Thought and Action: A Social Cognitive Theory.

[29] Bandura A. (1997). Self-efficacy: The Exercise of Control.

[30] G*Power 3.1. http://www.gpower.hhu.de/en.html.

[31] Gao Z., Pope Z., Lee J., Stodden D., Roncesvalles N., Pasco D., Huang C.C., Feng D. (2017). Impact of exergaming on young children’s school day energy expenditure and moderate-to-vigorous physical activity levels. J. Sport Health Sci..

[32] Evenson K.R., Catellier D.J., Gill K., Ondrak K.S., McMurray R.G. (2008). Calibration of two objective measures of physical activity for children. J. Sport Sci..

[33] Love R., Adams J., Atkin A., Van Sluijs E. (2019). Socioeconomic and ethnic differences in children’ s vigorous intensity physical activity: A cross-sectional analysis of the UK Millennium Cohort Study. BMI Open.

[34] Sallis J.F., Prochaska J.J., Taylor W.C., Hill J.O., Geraci J.C. (1999). Correlates of physical activity in a national sample of girls and boys in grades 4 through 12. Health Psychol..

[35] Harter S. (1978). Effectance motivation reconsidered. Hum. Dev..

[36] Trost S.G., Pate R.R., Saunders R., Ward D.S., Dowda M., Felton G. (1997). A prospective study of the determinants of physical activity in rural fifth-grade children. Prev. Med..

[37] Brustad R.J. (1993). Who will go out and play? Parental and psychological influences on children’s attraction to physical activity. Ped. Exerc. Sci..

[38] Luczynska G., Pena-Pereira F., Tobiszewski M., Namiesnik J. (2018). Expectation-Maximization model for substitution of missing values characterizing greenness of organic solvents. Molecules.

[39] Richardson J. (2011). Eta squared and partial eta squared as measures of effect size in educational research. Educ. Res. Rev..

[40] Nunnally J.C. (1978). Psychometric Theory.

[41] Gao Z., Zeng N., Pope Z., Wang R., Yu F. (2019). Effects of exergaming on motor skill competence, perceived competence, and physical activity in preschool children. J. Sport Health Sci..

[42] Gao Z., Chen S. (2014). Are field-based exergames useful in preventing childhood obesity? A systematic review. Obes. Rev..

[43] Gao Z., Chen S., Pasco D., Pope Z. (2015). Effects of active video games on physiological and psychological outcomes among children and adolescents: A meta-analysis. Obes. Rev..

[44] Staiano A.E., Beyl R.A., Hsia D.S., Katzmarzyk P.T., Newton R.L. (2017). Twelve weeks of dance exergaming in overweight and obese adolescent girls: Transfer effects on physical activity, screen time, and self-efficacy. J. Sport Health Sci..

[45] Gao Z. (2017). Fight fire with fire: Promoting physical activity and health through active video games. J. Sport Health Sci..

[46] Murphy E.C.S., Carson L., Neal W., Baylis C., Donley D., Yeateeerrr R. (2009). Effects of an exercise intervention using Dance Dance Revolution on endothelial function and other risk factors in overweight children. Int. J Pediat. Obes..

[47] Viana R.B., Vancini R.L., Vieira C.A., Gentil P., Campos M.H., Andrade M.S., de Lira C.A.B. (2018). Profiling exercise intensity during the exergame Hollywood Workout on XBOX 360 Kinect (R). Peer J..

[48] Peng W., Crouse J.C., Lin J.H. (2013). Using active video games for physical activity promotion: A systematic review of the current state of research. Health Educ. Behav..

[49] Lopez-Sanchez G.F., Diaz-Suarez A., Radziminski L., Jastrzebski Z. (2017). Effects of a 12-week-long program of vigorous-intensity physical activity on the body composition of 10-and 11-year-old children. J. Hum. Sport Exerc..

[50] Skrede T., Stavnsbo M., Aadland E., Aadland K.N., Anderssen S.A., Resaland G.K., Ekelund U. (2017). Moderate-to-vigorous physical activity, but not sedentary time, predicts changes in cardiometabolic risk factors in 10-y-old children: The Active Smarter Kids Study. Am. J. Clin. Nutr..

[51] Elmesmari R., Reilly J.J., Martin A., Paton J.Y. (2017). Accelerometer measured levels of moderateto-vigorous intensity physical activity and sedentary time in children and adolescents with chronic disease: A systematic review and meta-analysis. PLoS ONE.

[52] Flynn R.M., Staiano A.E., Beyl R., Richert R.A., Wartella E., Calvert S.L. (2018). The influence of active gaming on cardiorespiratory fitness in Black and Hispanic youth. J. Sch. Health.

[53] Calfas K.J., Sallis J.F., Oldenburg B., French M. (1997). Mediators of change in physical activity following an intervention in primary care: PACE. Prev. Med..

[54] Nichols J.F., Wellman E., Caparosa S., Sallis J.F., Calfas K.J., Rowe R. (2000). Impact of a worksite behavioral skills intervention. Am. J. Health Promot..

[55] Dowda M., Dishman R., Pfeiffer K., Pate R. (2007). Family support for physical activity in girls from 8th to 12th grade in South Carolina. Prev. Med..

[56] Hohepa M., Scragg R., Schofield G., Kolt G., Schaaf D. (2007). Social support for youth physical activity: Importance of siblings, parents, friends and school support across a segmented school day. Int. J. Behav. Nutri. Phys. Act..

[57] Baranowski T. (2017). Exergaming: Hope for future physical activity? or blight on mankind?. J. Sport Health Sci..

